# Urgent initiation of hemodialysis versus peritoneal dialysis in severe hyperkalemia: a prospective study

**DOI:** 10.1093/ckj/sfaf343

**Published:** 2025-11-06

**Authors:** Luz Alcantar-Vallin, José J Zaragoza, Francisco O Ruíz-Ochoa, J Emilio Sánchez-Alvarez, Gael Chávez-Alonso, Yulene Navarro-Viramontes, Narda Ramírez-Cortes, Fernando-Cruz Aragon, Karla Y Vargas-Langarica, Guillermo Navarro-Blackaller, Ramón Medina-González, Alejandro Martínez Gallardo-González, Juan A Gómez-Fregoso, Eduardo Mendoza-Gaitán, Guillermo García-García, Jonathan S Chávez-Iñiguez

**Affiliations:** Nephrology Department, Hospital Civil de Guadalajara Fray Antonio Alcalde, Guadalajara, Jalisco, Mexico; University of Guadalajara Health Sciences Center, Guadalajara, Jalisco, Mexico; Intensive Care Unit, Hospital H+ Queretaro, Queretaro, Mexico; Nephrology Department, Hospital Civil de Guadalajara Fray Antonio Alcalde, Guadalajara, Jalisco, Mexico; University of Guadalajara Health Sciences Center, Guadalajara, Jalisco, Mexico; Hospital Universitario Central de Asturias, Oviedo, Spain; Nephrology Department, Hospital Civil de Guadalajara Fray Antonio Alcalde, Guadalajara, Jalisco, Mexico; Nephrology Department, Hospital Civil de Guadalajara Fray Antonio Alcalde, Guadalajara, Jalisco, Mexico; University of Guadalajara Health Sciences Center, Guadalajara, Jalisco, Mexico; Nephrology Department, Hospital Civil de Guadalajara Fray Antonio Alcalde, Guadalajara, Jalisco, Mexico; University of Guadalajara Health Sciences Center, Guadalajara, Jalisco, Mexico; Nephrology Department, Hospital Civil de Guadalajara Fray Antonio Alcalde, Guadalajara, Jalisco, Mexico; University of Guadalajara Health Sciences Center, Guadalajara, Jalisco, Mexico; Nephrology Department, Hospital Civil de Guadalajara Fray Antonio Alcalde, Guadalajara, Jalisco, Mexico; University of Guadalajara Health Sciences Center, Guadalajara, Jalisco, Mexico; Nephrology Department, Hospital Civil de Guadalajara Fray Antonio Alcalde, Guadalajara, Jalisco, Mexico; University of Guadalajara Health Sciences Center, Guadalajara, Jalisco, Mexico; Nephrology Department, Hospital Civil de Guadalajara Fray Antonio Alcalde, Guadalajara, Jalisco, Mexico; Nephrology Department, Hospital Civil de Guadalajara Fray Antonio Alcalde, Guadalajara, Jalisco, Mexico; University of Guadalajara Health Sciences Center, Guadalajara, Jalisco, Mexico; Nephrology Department, Hospital Civil de Guadalajara Fray Antonio Alcalde, Guadalajara, Jalisco, Mexico; University of Guadalajara Health Sciences Center, Guadalajara, Jalisco, Mexico; Nephrology Department, Hospital Civil de Guadalajara Fray Antonio Alcalde, Guadalajara, Jalisco, Mexico; University of Guadalajara Health Sciences Center, Guadalajara, Jalisco, Mexico; Nephrology Department, Hospital Civil de Guadalajara Fray Antonio Alcalde, Guadalajara, Jalisco, Mexico; University of Guadalajara Health Sciences Center, Guadalajara, Jalisco, Mexico

**Keywords:** chronic hemodialysis, ESRD, hyperkalemia, peritoneal dialysis, uremic toxins

## Abstract

**Introduction:**

In patients with incident end-stage kidney disease (ESKD), hyperkalemia (HyperK) is a common indication for initiating kidney replacement therapy (KRT). Hemodialysis (HD) and peritoneal dialysis (PD) both effectively reduce serum potassium, but HD is often considered superior due to its perceived faster efficiency. However, evidence supporting this perception remains limited. We hypothesized that HD and PD would be equally effective for the management of severe HyperK during hospitalization.

**Methods:**

We conducted a prospective cohort study at the Nephrology Department of Hospital Civil de Guadalajara. Consecutive, dialysis-naïve patients hospitalized with ESKD and severe HyperK (serum potassium >6.5 mEq/L at admission) between 2022 and 2024 were included. The modality of KRT (HD vs PD) was determined by the treating nephrology team. The primary outcome was the trajectory of serum potassium reduction between groups. Secondary outcomes included daily potassium trajectory, catheter dysfunction, length of stay and mortality.

**Results:**

Eighty-two patients were included: 34 initiated PD, 37 HD and 11 received conservative management. Baseline demographic and clinical characteristics were similar across groups (*P* > .05). Median age was 65 years [interquartile range (IQR) 53–74], with diabetes in 33% and hypertension in 53%. Median admission potassium was 6.99 mEq/L (6.7–7.6), serum creatinine 15.9 mg/dL (11.5–23.1) and estimated glomerular filtration rate 2.91 mL/min/1.73 m² (1.80–4.09). The PD group underwent a mean of 45 (±15) exchanges during hospitalization, and the HD group received 4.6 (±1) sessions. Serum potassium decreased similarly in both groups (*P* > .05), with substantial reductions on Day 1 (PD 6.03 mEq/L; HD 5.90 mEq/L) and stabilization by Day 5 through Day 15. Catheter dysfunction occurred in 11% of patients, with similar rates between groups, hospitalization median was 5 days (IQR 3–8) and 12-month mortality was 26.8%, without differences between modalities.

**Conclusions:**

In this prospective cohort of ESKD patients with severe HyperK, both PD and HD achieved comparable potassium reduction and clinical outcomes, supporting PD as an effective alternative for urgent-start KRT.

KEY LEARNING POINTS **What was known:**

- Hyperkalemia (HyperK) is a leading indication for initiating kidney replacement therapy (KRT) in patients with end-stage kidney disease (ESKD), and hemodialysis (HD) has traditionally been considered the fastest and most effective method.
- Evidence directly comparing the efficacy of HD and peritoneal dialysis (PD) for urgent treatment of severe HyperK is scarce.
- Clinical perceptions often favor HD over PD despite limited data supporting this assumption.

**This study adds:**

- In a prospective cohort of incident ESKD patients with severe HyperK, PD and HD achieved comparable reductions in serum potassium, starting from the first day of therapy.
- Both modalities maintained potassium within safe ranges after Day 5 of treatment, with no recurrence of severe HyperK.
- Mortality and clinical outcomes at 12 months were similar between PD and HD, challenging the belief that HD is inherently superior.

**Potential impact:**

- PD can be considered a viable and effective alternative to HD for urgent treatment of severe HyperK in ESKD patients.
- These results may broaden access to urgent KRT in centers where HD is not immediately available.
- Findings support the design of randomized clinical trials to confirm the equivalence of PD and HD in this setting, potentially influencing future guidelines.

## INTRODUCTION

Kidney function is the variable most strongly associated with potassium excretion [1, 2]. As the glomerular filtration rate (GFR) deteriorates, the risk of hyperkalemia (HyperK) increases, placing individuals with end-stage kidney disease (ESKD) at the highest risk for this complication [3]. In addition to estimated glomerular filtration rate (eGFR), comorbidities commonly associated with chronic kidney disease (CKD), such as diabetes [4], heart failure [4] and the medications used in their treatments, are significant contributors that interact with serum potassium levels, markedly increasing them [5]. HyperK increases healthcare costs [6], contributes to CKD progression [7] and elevates the risk of mortality [8]. The Kidney Disease: Improving Global Outcomes (KDIGO) guidelines identify HyperK as one of the most critical complications of CKD, when HyperK is refractory to medical therapy, a 2B recommendation is issued to initiate kidney replacement therapy (KRT) [9]. Furthermore, normalization of serum potassium levels has been associated with a reduced risk of mortality [10]. In individuals with incident ESKD, HyperK is a common indication for initiating KRT [11]. Hemodialysis (HD) or peritoneal dialysis (PD) effectively lower serum potassium levels, essentially removing it from the circulation through diffusion [12]. In HD, using a synthetic membrane, and in PD, utilizing the peritoneum as a natural filter, dialysis fluids with potassium concentrations lower than those in serum are prescribed in both modalities, facilitating potassium exchange and its rapid removal [13, 14]. Although both modalities are effective, the nephrology community generally perceives HD as more efficient and rapid than PD for treating HyperK in newly diagnosed ESKD patients requiring urgent start KRT [3, 14]; however, the supporting evidence for this perception remains limited and underexplored. Furthermore, in this clinical scenario, where urea

reduction has been shown to be comparable between the two KRT modalities [15] and considering that potassium clearance kinetics are similar to those of urea, HD and PD are expected to be equally effective in managing severe HyperK during hospitalization in this vulnerable population. To address this knowledge gap, we conducted a cohort study involving incident ESKD patients requiring urgent start KRT due to severe HyperK, in which serum potassium trajectories were compared during hospitalization with either HD or PD, as well as over an extended follow-up period.

## MATERIALS AND METHODS

This prospective cohort study was conducted in the Department of Nephrology at Hospital Civil de Guadalajara, Mexico. It is a tertiary referral academic institution dedicated to the care of vulnerable populations in western Mexico. The study included incident and dialysis naïve patients who were consecutively hospitalized with severe ESKD. ESKD was defined as late-stage CKD (grade 5), based on KDIGO guidelines, with an eGFR <15 mL/min/1.73 m^2^ as calculated by the Chronic Kidney Disease Epidemiology Collaboration equation [9]. Consecutive incident adult patients aged >18 years with ESKD hospitalized for severe HyperK (serum potassium >6.5 mEq/L) and requiring urgent initiation of KRT were enrolled between May 2022 and October 2024.

This cohort is independent of our previously published study, which analyzed outcomes of dialysis initiation in patients with severe, symptomatic kidney failure [15]. The present study focused exclusively on patients with severe HyperK as the indication for urgent KRT initiation. There was no overlap in patient populations between the two cohorts. Severe HyperK was defined in accordance with criteria used in prior studies [3, 16, 17]. Serum potassium levels were measured every 24 h via venous puncture, using the ion selective method by the Abbott Alinity analyzer in the hospital’s central laboratory. Patients who declined initiation of KRT were categorized as “conservative” and received all available medical treatments for HyperK, excluding KRT. Exclusion criteria to study initiation include prior history of chronic dialysis and incomplete clinical data at admission. Patients with fewer than two serum potassium measurements or insufficient data to assess mortality, catheter dysfunction or dialysis modality at the end of follow-up were excluded.

All patients received medical treatment for HyperK alongside KRT, which was subsequently adjusted based on the trajectory of serum potassium levels. In general, medications associated with HyperK were temporarily discontinued, including renin–angiotensin–aldosterone system inhibitors, β-blockers, analgesics and certain antibiotics such as trimethoprim. Congestion was defined clinically by the presence of dyspnea and peripheral edema, together with ultrasound criteria of pulmonary congestion (>6 B-lines) or venous congestion [Venous Excess Ultrasound (VExUS) grade ≥2]. Patients with hypovolemia or dehydration received volume resuscitation. Polarizing solutions containing glucose and insulin were administered, along with furosemide for volume overload, intravenous sodium bicarbonate for severe metabolic acidosis, and nebulized salbutamol. Intestinal potassium-binding resins, including sodium polystyrene sulfonate, calcium polystyrene sulfonate, patiromer and sodium zirconium cyclosilicate, were not available at our center during the study period.

For the initiation of KRT, the nephrology team evaluates the eligibility of either HD or PD based on individual clinical criteria, considering absolute contraindications for PD, including loss of the peritoneal integrity, peritoneal adhesions, uncorrectable hernia, stomas, diaphragmatic defects or inability to administer treatment at home [18]. The benefits and potential complications of both HD and PD are clearly explained to patients and their families to facilitate informed decision-making regarding the most appropriate modality. Both HD and PD catheters are routinely inserted within an average of 6 h from hospital admission. Our center is equipped to initiate either modality at any time of the day. The hospital offers maintenance programs for both PD and HD, with treatment costs fully covered by the institution. The HD tunneled vascular catheter used was the SMART by EBIME^®^, a high-flux polyurethane device measuring 14.5 French in diameter and 28 cm in length. Catheter placement was performed by a nephrology resident under the supervision of an experienced nephrologist, using ultrasound guidance, preferably via the right internal jugular vein. Correct catheter placement was confirmed by visualizing the tip within the right atrium, followed by immediate initiation of an HD session [19]. HD sessions were scheduled within 3–4 h after catheter placement and conducted using Bellco Formula Therapy^®^ machines. Prescriptions were individualized based on clinical needs; however, each session typically lasted 3 h 30 min. A high-flux Diapes BLS 819 filter was selected according to body surface area (1.7–1.9 m^2^), with a blood flow of 350 mL/min and dialysis flow of 500 mL/min. Dialysate electrolyte concentrations were 136 mEq/L for sodium, 2.0 mEq/L for potassium and 2.0 mEq/L for calcium. Ultrafiltration was adjusted based on the presence of volume overload, and anticoagulation was provided with heparin. Following discharge, HD was prescribed incrementally, beginning with two sessions per week [20].

The peritoneal catheter used was a Tenckhoff-type, composed of silicone, measuring 42 cm in length, equipped with two Biosil^®^ cuffs, a titanium connector and a Y-shaped transfer line. The PD catheter was inserted by two nephrology residents under the supervision of a nephrologist. The catheter was inserted percutaneously, with prophylactic ceftriaxone, local anesthesia using 2% lidocaine, and, in some patients, conscious sedation with dexmedetomidine. Through a paramedian incision located 3–4 cm from the umbilical scar, a straight silicone catheter was inserted in the right pelvic cavity under ultrasound guidance. One cuff was positioned within the rectus abdominis muscle, and the other was placed in the subcutaneous tissue. To ensure catheter functionality, PD was initiated immediately after insertion, beginning with three manual exchanges using an input–output technique. An initial fill volume of 1500 mL was used for the first manual exchange in all patients. This was followed by 30 automated exchanges using Pacifica^®^ machine, each with a dwell time of 1.5 h, which continued over a 2- to 3-day period and was further modulated according to clinical needs [21]. PiSA^®^ brand peritoneal dialysis bags contain varying glucose concentrations to establish an osmotic gradient, with selection guided by the presence of vascular congestion. The dialysis solutions contained potassium 0 mEq/L, sodium 132 mEq/L, calcium 3.5 mEq/L, magnesium 0.5 mEq/L, chloride 96 mEq/L and lactate 40 mEq/L, and had a pH range of 5.0–5.6. Following hospital discharge, patients and their families received training and were enrolled in the institutional maintenance PD program, utilizing either an automated or manual modality. Patients selected either continuous ambulatory peritoneal dialysis (CAPD) or automated peritoneal dialysis (APD) based on individual preferences and capabilities. Cycle prescriptions were incremental, consisting of two to three exchanges per day for CAPD or three to four exchanges per day for APD [22]. Enrollment in these programs typically require approximately 3–4 weeks, during which patients are readmitted to the hospital two to three times for intermittent PD based on clinical requirements. Throughout this period, patients and their families underwent training to perform PD at home. The training was delivered through practical sessions led by nurses and social workers within the CAPD program. Upon completion of training, theoretical and practical assessments are conducted to evaluate whether patients and their families are prepared to perform PD exchanges safely.

Patients in the HD group underwent HD on an alternate-day schedule, adjusted to clinical status and serum potassium levels. By contrast, patients in the PD group received continuous treatment, performing multiple daily exchanges throughout hospitalization.

### Data acquisition

Demographic and clinical variables, comorbidities, serum electrolyte levels and acid–base status were recorded. The trajectory of key variables was documented during the first 15 days of hospitalization to evaluate the effectiveness of urgent KRT. The variables evaluated included mortality, length of hospital stay and catheter dysfunction. Patients were followed up for 12 months from the initiation of KRT, with clinical status and survival verified through medical consultations, telephone interviews or electronic medical records. PD catheter dysfunction was defined as the inability of the catheter outflow to sustain further dialysis due to impaired drainage from the peritoneal cavity, necessitating a procedural intervention [23]. HD discontinuation was defined as the transition to PD owing to the inability to continue HD. Catheter-related infections associated with HD were documented in hospital medical records.

### Primary and secondary outcomes

The primary outcome was the trajectory of HyperK in the PD and HD groups. Secondary outcomes included the daily trajectory of serum potassium levels, patient mortality, catheter dysfunction and dialysis modality at the end of the follow-up period. Participants received no financial compensation. This study was funded through a grant provided by the Secretaria de Salud Jalisco y el and the Consejo Nacional de Ciencia, Humanidades y Tecnología (CONAHCYT). Written informed consent was obtained from all participants. This study was approved by the Hospital Civil de Guadalajara Fray Antonio Alcalde Institutional Review Board (CEI 188/22). The study adhered to the Strengthening the Reporting of Observational Studies in Epidemiology guidelines [24] and the Reporting of Studies Conducted using the Observational Routinely Collected Health Data statement [25]. The principles of the Declaration of Helsinki for medical research involving human subjects were followed to ensure ethical standards and methodological transparency.

### Statistical analysis

The distribution of quantitative variables was assessed visually using histograms, along with the Shapiro–Wilk test and tests for skewness and kurtosis, to confirm non-normality. Dry weight was excluded due to more than 30% missing values. Continuous variables were assessed for normality using the Shapiro–Wilk test. Variables with normal distribution are presented as mean ± standard deviation (SD), while those with non-normal distribution are presented as median [interquartile range (IQR)]. Categorical variables are reported as counts and percentages.

Patient groups were classified based on treatment modalities. Differences in categorical variables between groups were analyzed using the chi-square test or Fisher’s exact test, as appropriate. Continuous variables were compared using the Kruskal–Wallis test for three groups (conservative, PD, HD) and the Mann–Whitney U test for comparisons between PD and HD. A complete-case analysis was performed for all statistical tests.

The progression of serum potassium was illustrated using box plots from Day 1 to 10 of hospitalization, comparing between both KRT modalities. Confidence interval (CI) plots were also used, and a Student’s *t*-test was applied for each day to compare values between groups. Additionally, the Mann–Whitney U test was employed to compare daily median potassium levels, while a Student’s *t*-test was performed to assess daily mean potassium differences between groups. A paired *t*-test was conducted to compare potassium levels on Day 1 with subsequent days (i.e. Day 1 vs Day 2, Day 1 vs Day 3, etc.) for each group independently. A time-series line plot of serum potassium was generated to visually depict its reduction by group, displaying the mean as a point estimate with 95% CIs. A Kaplan–Meier analysis was performed to estimate the probability of achieving normokalaemia (serum potassium <5.5 mEq/L) over time by group, with comparisons conducted using the log-rank test. Additionally, a linear regression model was applied with daily serum potassium as the dependent variable, using a random-effects approach with a generalized least squares estimator to produce a matrix-weighted average of the between- and within-group effects. Because serum creatinine (sCr) and eGFR are collinear, they were not entered simultaneously. Multicollinearity was assessed with variance inflation factors. The primary model adjusted for sCr; a prespecified sensitivity analysis replaced sCr with eGFR (variables centered/standardized). Finally, Kaplan–Meier survival estimates were calculated for a 12-month follow-up period. Statistical significance was defined as a *P*-value <.05. Data were analyzed using Stata version 16.1 (StataCorp, College Station, TX, USA).

## RESULTS

**Figure 1:**
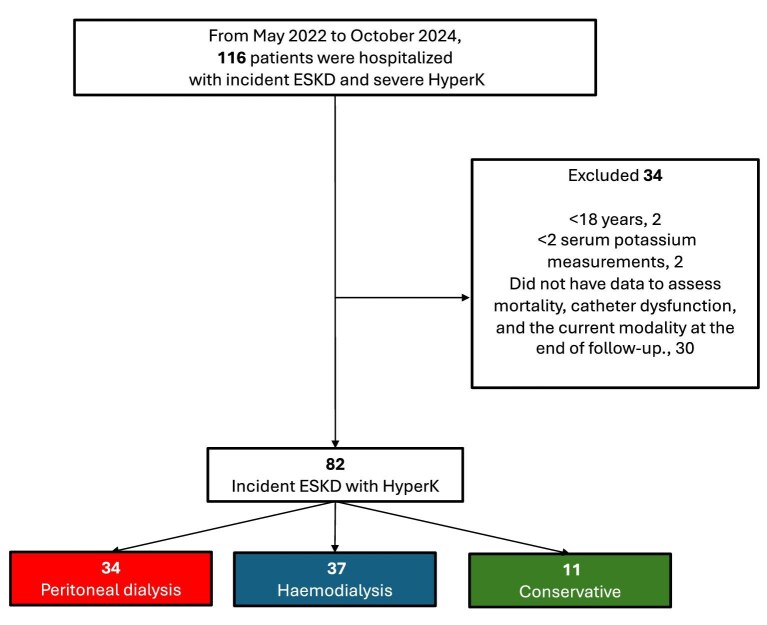
Flowchart of study cohort.

Between May 2022 and October 2024, 116 patients were hospitalized with ESKD and severe HyperK; 34 patients were excluded for not meeting the inclusion criteria. The final cohort included 82 patients with ESKD and severe HyperK: 34 were allocated to the PD group, 37 to the HD group and 11 to the conservative group, as shown in Fig. 1.

### Demographic and clinical characteristics

Table 1 summarizes the demographic and clinical characteristics of the entire cohort. In general, these characteristics were consistent with those typically observed in patients with incident ESKD and did not significantly differ between the study groups (*P* > .05). Among the most relevant findings, the mean age was 50 years (35–64), with diabetes and hypertension present in 33% and 53% of patients, respectively. The median hemoglobin level was 7.0 g/dL (5.48–9.16), serum potassium was 6.99 mEq/L (6.70–7.60) and sCr was 15.9 mg/dL (11.5–23.1), with an eGFR of 2.91 mL/min/1.73 m^2^ (1.80–4.09). Additionally, 25% of patients presented vascular congestion at admission. Diuretic treatment was administered to 70% of patients, being more common in the PD group (32%) compared with the HD group (28%) (*P* = .048). Catheter dysfunction occurred in 11% of patients and was similar between the groups initiating KRT. The median length of hospitalization was 5 days (3–8), and 22 patients (26.8%) died during hospitalization (Table 1).

**Table 1:** Baseline demographics and clinical characteristics of patients with ESKD and severe HyperK according to treatment modality at admission.

Variable	Conservative (*n* = 11)	PD (*n* = 34)	HD (*n* = 37)	Total (*n* = 82)	*P*	*P* (PD–HD)
Baseline characteristics						
Age (years)	50.09 (34.77–63.98)	50.32 (35.44–63.87)	49.95 (36.12–64.15)	50.10 (35.00–64.00)	.192	.112
Male sex, *n* (%)	9 (81.8)	17 (50)	23 (62.1)	49 (59.7)	.156	.345
Weight (kg)	76.3 (56–88)	70 (63–74)	69.5 (59.5–76.5)	70 (61.6–76.1)	.359	.869
Height (m)	1.7 (1.62–1.77)	1.63 (1.6–1.68)	1.65 (1.56–1.7)	1.65 (1.6–1.7)	.243	.822
BMI	26.4 (35.9–29.7)	25.1 (23.4–27.8)	25.4 (22.1–27.5)	25.5 (22.6–28)	.743	.934
Systolic pressure (mmHg)	121 (91–144)	140 (130–160)	140 (118–160)	140 (121–156)	.097	.556
Diastolic pressure (mmHg)	77 (64–89)	80 (69–94)	80 (70–95)	80 (69–94)	.727	.953
Heart rate	89 (70–100)	91 (80–104)	85 (78–100)	86 (75–100)	.533	.580
Respiratory rate	20 (17–24)	20 (18–22)	20 (18–21)	20 (18–22)	.979	.976
Diabetes mellitus	7 (70)	11 (32.35)	15 (42.86)	33 (41.77)	.115	.458
Hypertension	6 (60)	24 (7.59)	23 (65.71)	53 (67.09)	.799	.797
Heart disease	1 (10)	4 (11.76)	1 (2.86)	6 (7.59)	.330	.198
Polycystic kidney disease	0	0	1 (2.86)	1 (1.27)	1.000	1.000
SpO2	95 (94–97)	94 (92–96)	95 (92–97)	95 (92–97)	.449	.397
Hemoglobin	6.88 (5.89–9.07)	6.93 (5.48–8.73)	7.53 (5.4–9.44)	7.0 (5.48–9.16)	.877	.769
Serum potassium	6.95 (6.72–7.61)	6.99 (6.70–7.60)	6.98 (6.71–7.59)	6.99 (6.70–7.60)	.186	.055
sCr	10.3 (7.92–27.1)	17.9 (11.7–28.4)	16.2 (11.6–20.3)	15.9 (11.5–23.1)	.124	.187
Urea	327 (194–412)	331 (236–379)	323 (273–405)	327 (248–404)	.956	.746
eGFR	4.46 (3.57–6.89)	2.63 (1.55–3.59)	2.87 (2.05–3.96)	2.91 (1.80–4.09)	.033^*^	.181
Total body water	43.7 (40.6–45)	33.5 (28.4–41.2)	36.3 (30.4–38.6)	35.4 (29.8–41.3)	.092	.549
% ECW	25 (13.3–28.8)	19.05 (3.5–30.2)	13.5 (2.95–26.6)	17.2 (3.5–27.2)	.548	.566
% LTM	67.4 (60.9–72.4)	51.8 (40.9–69.7)	57.0 (39.6–62.6)	57 (43–67.4)	.208	.934
% Fat	15.8 (15.6–28.1)	32.3 (15.4–42.8)	31.1 (22.5–42)	31 (16.4–40.1)	.199	.915
Urgent KRT	11 (100)	30 (90.91)	28 (75.68)	69 (90.79)	.573	.708
Vascular congestion	5 (45.45)	9 (28.13)	11 (33.33)	25 (32.89)	.573	.789
Diuretic treatment	10 (90.91)	32 (94.12)	28 (75.68)	70 (85.37)	.086	.048
GIK therapy	8 (72.73)	23 (67.65)	22 (59.46)	53 (64.63)	.696	.623
HD sessions during hospitalization	0	0	4.58 (1)	4.58 (1)		
PD exchanges during hospitalization	0	45 (15)	0	45 (15)		
Catheter dysfunction	0	4 (11.76)	5 (13.51)	11 (13.58)		1.000
Outcomes						
Days of hospital stay	4.5 (2–8)	4 (3–8)	6 (4–8.5)	5 (3–8)	.323	.185
Death in hospital	5 (45.45)	8 (23.53)	9 (24.32)	22 (26.83)	.340	1.000

Values are expressed as mean (SD) for normally distributed variables, or median (interquartile range) for non-normally distributed variables. The entry “4.58 (1)” represents mean (SD). Categorical variables are presented as counts (percentages).

BMI, body mass index; ECW, extracellular water; GIK, glucose–insulin–potassium; LTM, lean tissue mass.

Patients were divided into three treatment groups: conservative, PD and HD. Significant differences were observed in eGFR (mL/min/1.73 m^2^) among the group: 4.46 (3.57–6.89) for the conservative group, 2.63 (1.55–3.59) for PD and 2.87 (2.05–3.96) for HD (*P *= .033). Patients in the PD group underwent an average of 45 exchanges (SD ±15) during hospitalization, whereas those in the HD group underwent 4.58 sessions (SD ±1).

### Primary objective: serum potassium trajectories between PD and HD groups

**Figure 2:**
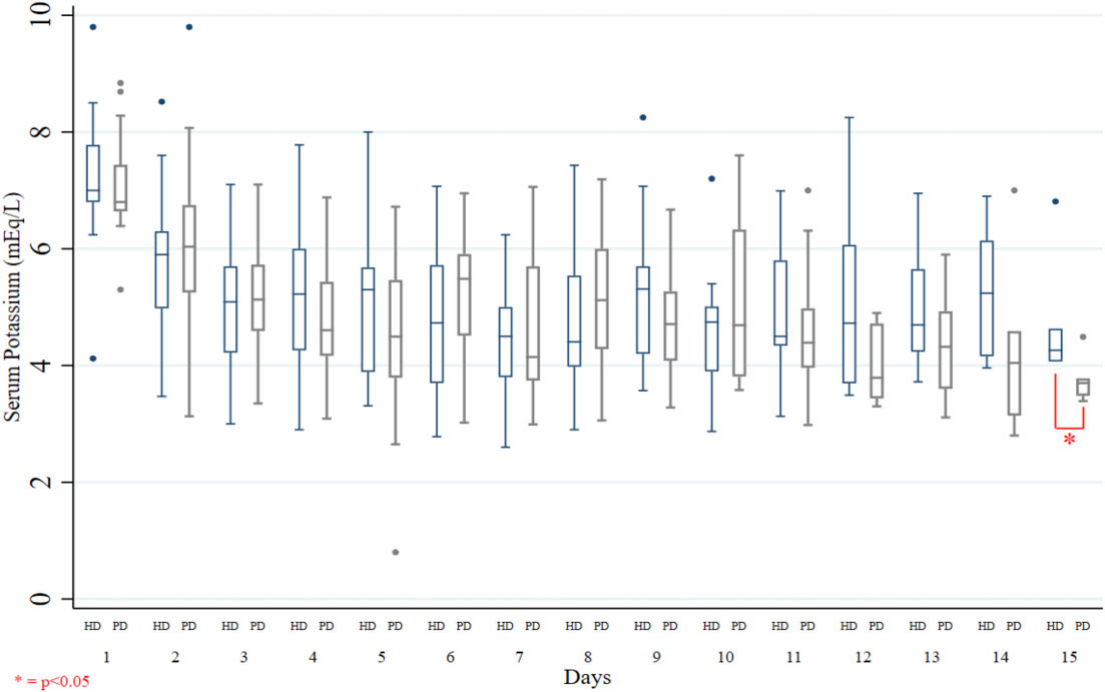
Box plot of serum potassium per day by groups. The box plot illustrates the daily trajectory of serum potassium levels from the initiation of HD or PD in patients with severe HyperK. The most marked decrease in serum potassium occurs after the first day of KRT. Subsequently, both groups exhibit a gradual decline, reaching near-normal levels by Days 4 to 5. From that point onward, serum potassium remains within the normal range through the end of the observation period. Notably, by Day 15, serum potassium levels are significantly lower in patients receiving PD compared with those undergoing HD.

The trajectories of serum potassium levels during hospitalization, stratified by treatment group, are presented in Table 2. Over the first 15 days, serum potassium levels declined similarly in both groups (*P* > .05). Initial serum potassium levels were 6.8 mEq/L (6.64–7.44) in the PD group and 7.0 mEq/L (6.8–7.78) in the HD group (*P* = .281). As shown in Fig. 2, serum potassium levels began to decline from the first day of KRT in both modalities [PD 6.03 mEq/L (5.25–6.75) and HD 5.90 mEq/L (4.98–6.30)] and continued to decrease steadily. By the fifth day, both groups approached normokalaemia with potassium levels of 4.74 mEq/L (4.17–5.69, *P* = .20 between both groups), as detailed in Table 2. Figure 2 also demonstrates a plateau in serum potassium levels, with no significant changes from Day 5 through Day 15. At Day 15, levels were 3.7 mEq/L (3.48–3.78) in the PD group and 4.26 mEq/L (4.07–4.63) in the HD group (*P* = .106).

**Table 2:** Serum potassium (mEq/L) trajectories per day by study groups.

Day	PD	HD	Total	*P*-value	*P*-value for *t*-test
1	6.8 (6.64–7.44)	7.0 (6.80–7.78)	6.99 (6.70–7.60)	.055	.281
2	6.03 (5.25–6.75)	5.90 (4.98–6.30)	6 (4.98–6.53)	.284	.267
3	5.13 (4.59–5.73)	5.09 (4.22–5.70)	5.11 (4.46–5.71)	.580	.624
4	4.60 (4.16–5.43)	5.22 (4.26–60)	4.74 (4.17–5.69)	.201	.179
5	4.49 (3.79–5.46)	5.3 (3.89–5.68)	4.8 (3.82–5.50)	.364	.228
6	5.48 (4.51–5.91)	4.73 (3.70–5.72)	5.18 (4.08–5.91)	.451	.417
7	4.50 (3.8–5.54)	4.50 (3.80–50)	4.32 (3.77–5.12)	.951	.790
8	5.12 (4.28–6)	4.40 (3.98–5.54)	4.9 (4.09–5.58)	.262	.359
9	4.71 (4.08–5.27)	5.32 (4.20–5.70)	4.91 (4.17–5.60)	.347	.370
10	4.69 (3.82–6.33)	4.74 (3.9–5.01)	4.69 (3.9–5.32)	.826	.560
11	4.39 (3.96–4.98)	4.5 (4.34–5.8)	4.5 (3.99–5.12)	.597	.642
12	3.79 (3.43–4.72)	4.72 (3.69–6.06)	4.24 (3.61–4.87)	.188	.106
13	4.32 (3.6–4.93)	4.69 (4.23–5.65)	4.65 (3.92–4.93)	.366	.312
14	4.04 (3.14–4.59)	5.24 (4.16–6.14)	4.41 (3.90–5.91)	.200	.223
15	3.7 (3.48–3.78)	4.26 (4.07–4.63)	4.07 (3.7–4.49)	.041^*^	.106

### Secondary objectives: variables associated with serum potassium variation, serum potassium trajectories in patients receiving conservative management, and determination of the risk of HyperK and long-term mortality

To identify variables associated with reductions in serum potassium levels, a linear regression model was performed. Age, sCr and eGFR were positively associated with these changes (*P* for all <.05), as shown in Table 3. In this model, which used HD as the reference group, PD was not significantly associated with a difference in daily serum potassium levels (Coeff: –0.327, 95% CI –0.722 to 0.068, *P *= .105).

**Table 3:** Linear regression model for daily serum potassium.

Serum potassium	Coeff.	LCI	UCI	*P*
Diuretic	–0.0725	–0.5902	0.4452	.784
Age	0.0266	0.0152	0.0380	<.001
Heart disease	–0.0636	–0.7752	0.6479	.861
sCr	0.1434	0.1219	0.1649	<.001
eGFR	0.1383	0.0872	0.2800	<.001
PD vs HD (Reference)	–0.3271	–0.7229	0.0686	.105

OR, odds ratio; LCI, lower confidence interval; UCI, upper confidence interval.

**Figure 3:**
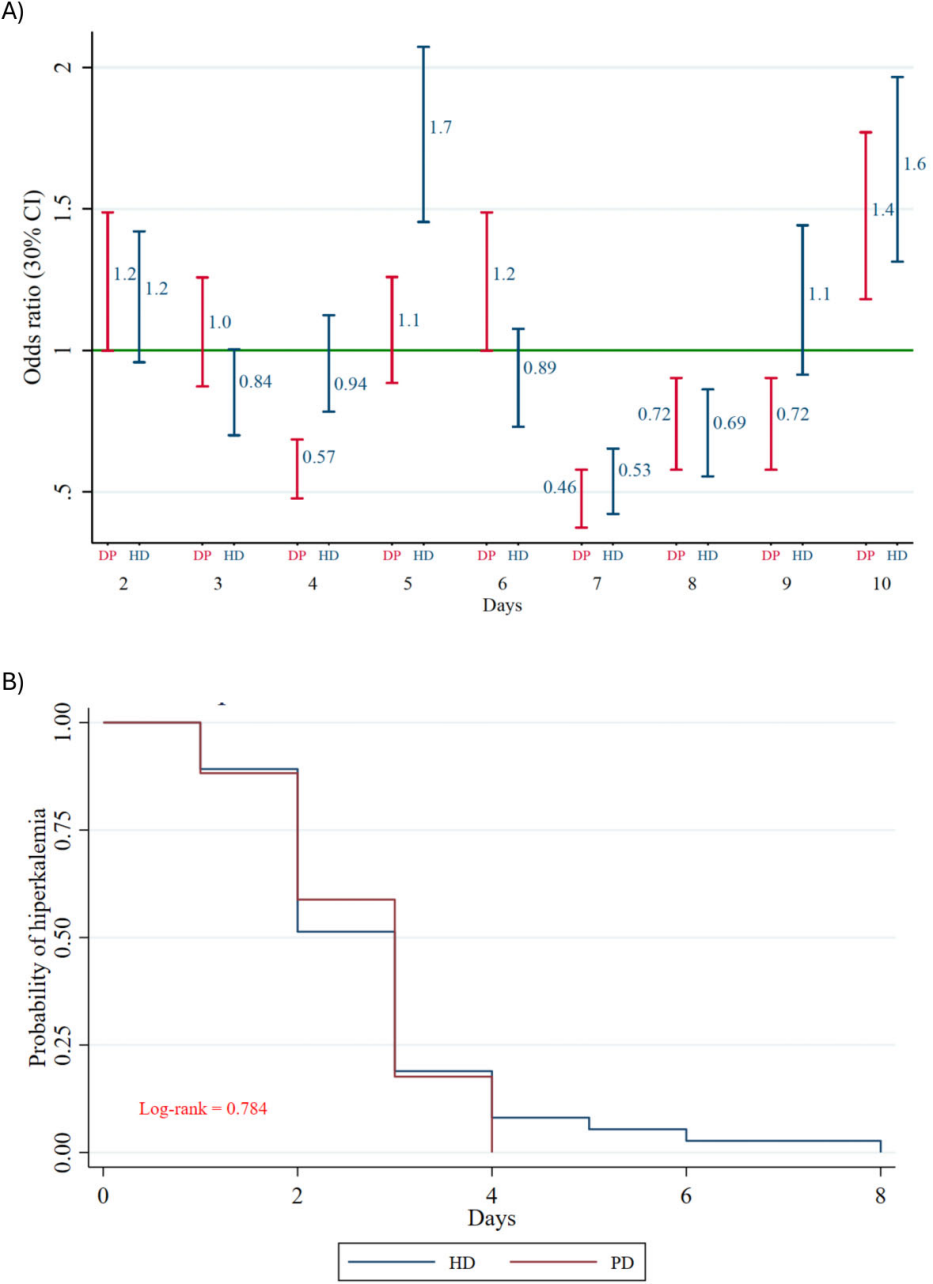
(**A**) Risk of staying hyperkalemic between PD and HD groups during the first 10 days of hospitalization and, (**B**) Kaplan–Meier estimates of the probability of HyperK. In (A), HD shows a more consistent trend toward increasing the likelihood of persistent HyperK, especially in Days 5 and 9. PD has a more stable effect, with some days of better control (Day 4). The clinical difference could be influenced by factors such as treatment frequency and ultrafiltration. In (B) The graph shows how the proportion of patients who continue to have HyperK decreases over time after starting KRT, whether HD or PD. At baseline (Day 0), all patients had HyperK, over the course of several days, HyperK resolved in both groups. By Day 4, most patients no longer had HyperK. After that point, very few patients still had HyperK. There is no significant difference between HD and PD in terms of the speed at which HyperK was resolved.

**Figure 4:**
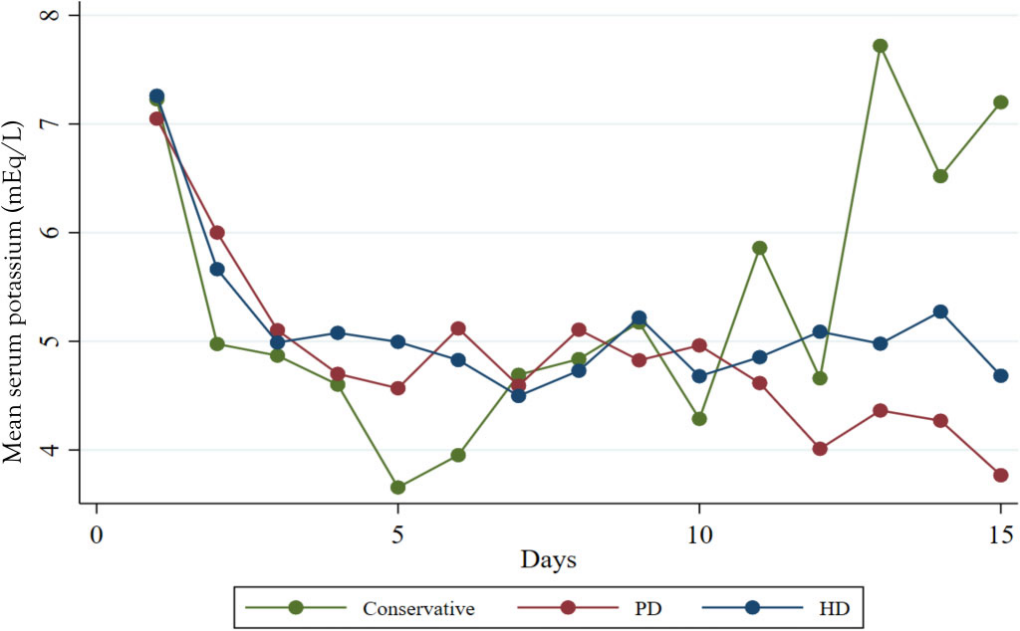
Serum potassium trajectory in conservative, PD and HD groups during the first 15 days. The time series graph shows the evolution of mean serum potassium levels (mEql/L) over 15 days in three groups of patients with severe HyperK: conservative treatment, PD and HD. All groups presented elevated potassium concentrations at the beginning of the follow-up (∼7 mmol/L). In the first days, a marked reduction in serum potassium levels was observed in all three groups, with a more pronounced and sustained decrease in the groups treated with PD and HD. From Day 5, potassium levels in the conservative group showed a tendency to instability, with significant peaks towards the end of the period (Days 10–15), again reaching dangerous values. In contrast, the PD and HD groups maintained greater stability in potassium levels, with progressively lower values. Notably, the PD and HD groups reached concentrations closer to normokalaemia by Day 15.

**Figure 5:**
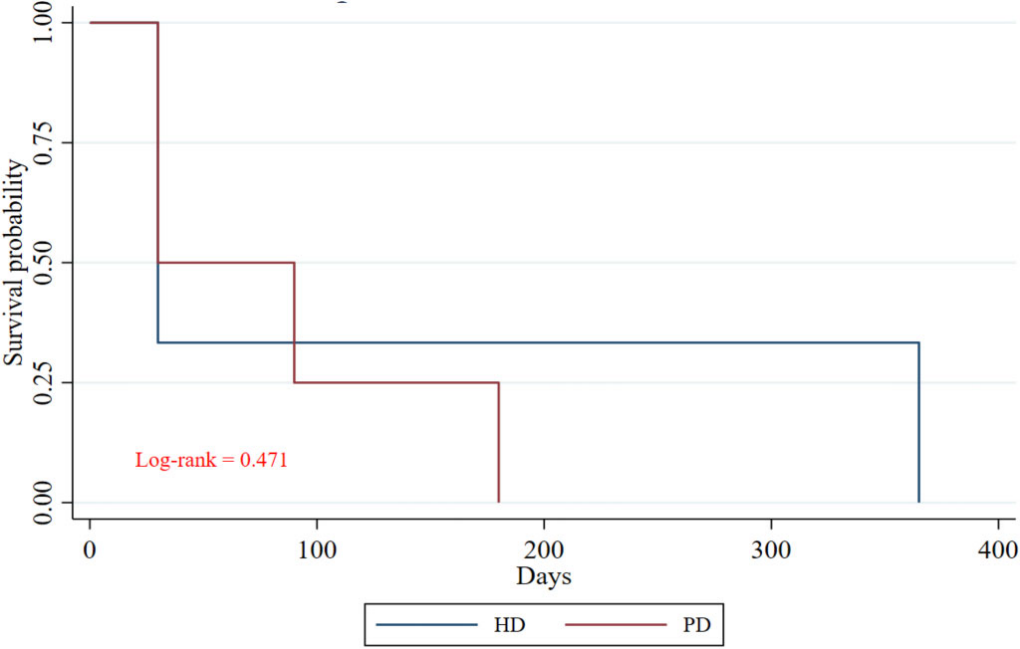
Survival estimates using Kaplan–Meier analysis in patients treated with conservative management, HD or PD. The curves show the survival probability over time for patients in the HD, PD and conservative management groups. All groups show an early decline in survival. Throughout long-term follow-up, survival was similar (log-rank test *P* = .749).

The daily risk of persistent HyperK was analyzed during the initial days of hospitalization for both groups. The risk decreased progressively during the first 3 days of KRT; by Days 4 and 5, it was significantly lower in the PD group. This difference may be attributed to the intermittent nature of HD sessions, during which serum potassium was measured before HD (Fig. 3A). Figure 3B illustrates that the probability of HyperK decreased to below 25% from Day 3 in both the PD and HD groups (log-rank *P *= .78), and this risk remained negligible throughout the hospital stay. Notably, the serum potassium trajectory in patients receiving conservative treatment declined over the first 5 days, then gradually rose to levels exceeding 5.0 mEq/L by Day 10 and subsequently increased further to severe HyperK (>6.5 mEq/L), comparable to levels observed at hospital admission. In contrast, patients on PD and HD did not experience a recurrence of severe HyperK (Fig. 4). The frequency of pharmacologic interventions for HyperK was comparable between the HD and PD groups (*P* > .05). Finally, long-term survival probabilities for the conservative, PD and HD groups are presented in Fig. 5. The survival curves show a visual trend towards lower survival in patients who received conservative management compared with those who initiated KRT. However, the overall survival among the three groups was not statistically significant over the 12-month follow-up period (log-rank *P* = .749). No significant survival differences were observed between the PD and HD modalities.

## DISCUSSION

**Figure 6:**
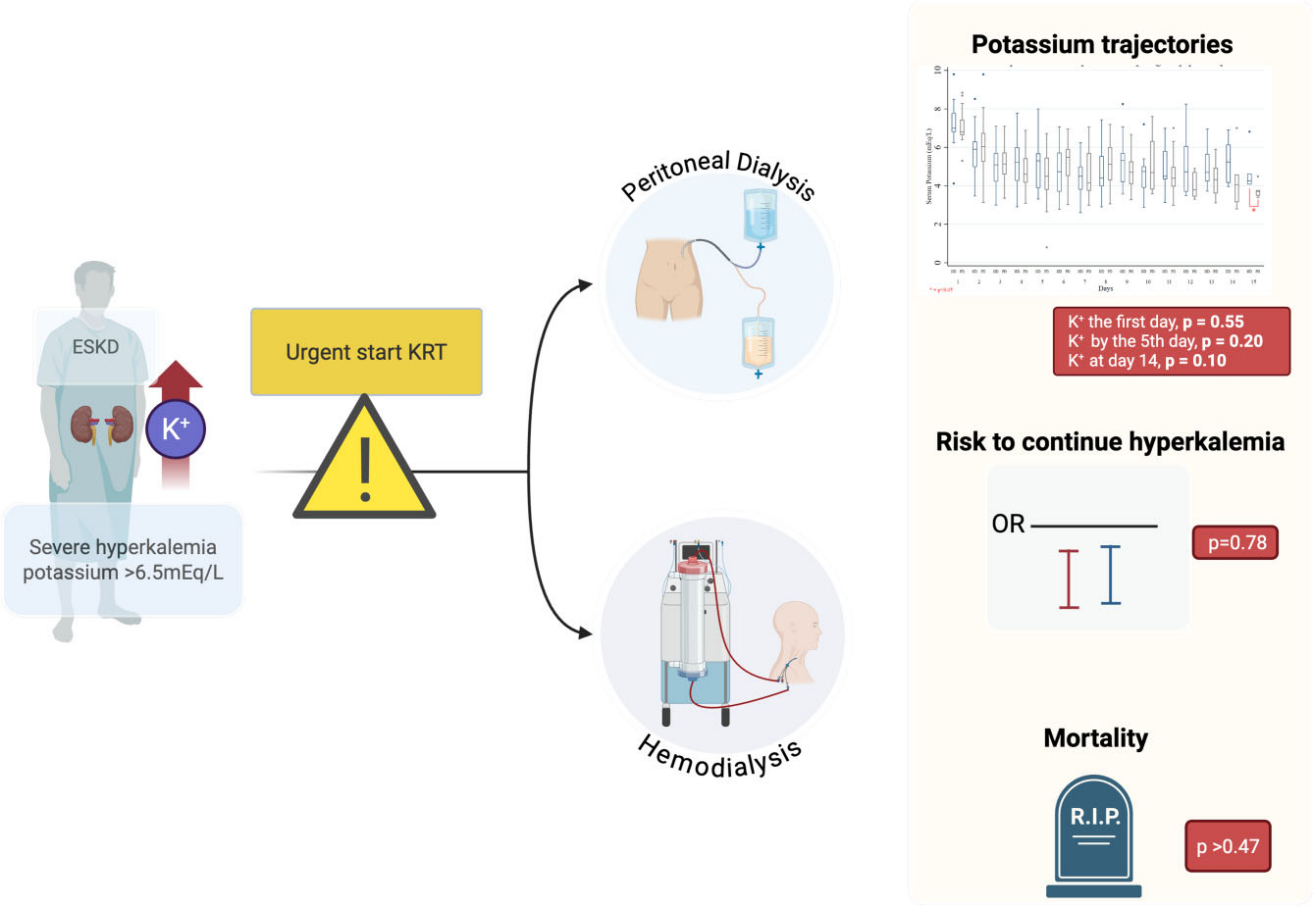
Central figure. OR, odds ratio.

In this prospective cohort of patients with ESKD and severe HyperK who urgently initiated KRT via PD or HD, serum potassium reduction was achieved at comparable rates between modalities. Serum potassium reductions began on the first day of treatment, achieving normokalaemia by Day 4 and stabilizing at safe levels through Day 15 of follow-up. Both PD and HD significantly reduced the risk of recurrent HyperK, an outcome frequently observed in patients managed conservatively (Fig. 6).

Serum potassium reduction in patients with incident ESKD and HyperK patients was found to be comparable between the PD and HD groups. This finding underscores the potential of PD to be as effective as HD in managing such emergencies. Traditionally, the nephrology community has favored HD in such clinical scenarios. We believe this assumption lacks evidence and should be explored further.

To date, this cohort appears to be unique, limiting opportunities for direct comparison with existing studies. These findings contribute to the expanding body of evidence supporting the effectiveness of PD in ESKD cases requiring urgent KRT initiation [26]. Previous analysis showed that symptomatic ESKD patients with urea levels exceeding 350 mg/dL, who required urgent initiation of PD or HD, exhibited comparable clinical courses during hospitalization and similar long-term outcomes, with effective correction of uremic symptoms and metabolic abnormalities [15].

The doses and modalities of KRT applied in this study were analogous to those commonly used in acute kidney injury (AKI) settings, where PD is urgently initiated to address metabolic complications such as HyperK. Published evidence indicates that serum potassium reduction is achieved in approximately 89% of PD cases [27]. The correction of HyperK in AKI using PD has demonstrated efficacy comparable to daily HD [28], slow low-efficiency dialysis [29] or continuous renal replacement therapy [30]. The efficacy of PD in reducing serum potassium can be attributed to the prescribed regimen, which includes potassium-free dialysis solutions administered during the initial 2–3 days. Typically, two exchanges of 6000 mL per day are performed using cycler machines, adjusted based on clinical requirements. This high-dose approach mirrors that used in AKI treatment [31], where correction of HyperK is also achieved within 3–4 days [29, 32]. Although early potassium trajectories were similar between modalities, the prolonged time connected to the PD cycler during the initial 48–72 h may reduce patient mobility and bedside autonomy, which carries practical implications for nursing care, ambulation and overall patient experience.

At our center, in cases of severe HyperK, HD sessions are prescribed using dialysate solutions containing 2.0 mmol/L potassium. This practice aligns with clinical recommendations and evidence indicating an increased risk of sudden cardiac death associated with potassium-free dialysate, which may create a steep potassium gradient and induce cardiac arrhythmias [33].

The secondary outcomes included the daily trajectory of serum potassium levels and mortality at the end of follow-up. One in four patients in our cohort died during follow-up, a high mortality rate that must be interpreted in the context of case severity. Previous reports have indicated that incident ESKD patients with HyperK are at an increased risk of mortality [34, 35]. This trend aligns with findings by Xu *et al*., where patients with HyperK >5.0 mEq/L exhibited a higher risk of mortality [34].

No significant difference in mortality was observed between the PD and HD groups. These results are comparable to those of a Finnish study, which reported a 1-year mortality rate of 29% without intergroup differences [26]. However, all patients in our cohort presented with severe HyperK, in contrast to the Virtanen *et al*. cohort, where the mean serum potassium level was 4.2 mmol/L (IQR 3.7–4.9), substantially lower than our cohort’s median of 6.99 mEq/L (IQR 6.7–7.6).

Patients who received conservative treatment demonstrated an initial reduction in serum potassium during the early days of hospitalization; however, levels subsequently increased to values comparable to those at admission. This outcome is anticipated, as the pharmacological interventions employed in these patients possess limited efficacy and are primarily intended for inpatient use. The variables found to influence serum potassium levels were age, sCr and eGFR. These findings are consistent with the pathophysiological mechanisms and risk factors for HyperK documented in the literature. Aging reduces the tubular capacity for potassium handling, and the kidneys play a critical role in potassium excretion, making individuals with lower eGFR more susceptible to HyperK [16, 36, 37].

Our findings should be interpreted considering their inherent limitations. One of the most pertinent limitations is the study design; as a cohort study, it can only generate hypotheses and identify associations. This study was not a randomized controlled trial, and the choice of dialysis modality was determined by the treating nephrologist according to clinical judgment. As such, the possibility of selection bias cannot be excluded, and our findings should be interpreted in this context. Numerous confounding variables may remain unaccounted for, despite adjustments made using multivariate analysis. Selection bias may be present among patients who received PD or HD; however, we believe it does not significantly affect our primary endpoint of serum potassium reduction or the associated clinical outcomes. The sample size is relatively small, potentially affecting statistical significance; however, it is comparable to previous cohorts that evaluated PD and urgently initiated HD. A further limitation is the strong collinearity between sCr and eGFR, which precluded their inclusion in the same model; to address this, sensitivity analyses were performed using one variable at a time. The exact time from hospital admission to KRT initiation was not systematically recorded; however, in our center, catheter placement and initiation of KRT are generally performed within the first 6 h after admission. Clinical symptoms and signs associated with hyperkalemia (e.g. muscle weakness, electrocardiographic abnormalities or arrhythmias) were not consistently available in patient records.

Strengths of this study include the enrollment of patients with severe HyperK, daily monitoring of serum potassium levels enabling a detailed temporal analysis of KRT effectiveness, and the consistent use of tunneled catheters in all HD patients.

The clinical significance of our findings is the hypothesis that PD may be a viable option in patients with ESKD and severe HyperK, and should not be excluded based on the unfounded belief that HD is inherently more effective in lowering serum potassium. These results can serve as a basis for future randomized clinical trials comparing both therapies in these complex clinical scenarios.

## CONCLUSIONS

In this cohort of patients with ESKD and severe HyperK who required urgent start of PD or HD, serum potassium levels decreased within the initial days of treatment and remained stable thereafter. Long-term mortality rates were comparable between the two groups.

## Data Availability

The files and data are in the physical and electronic archives of the Civil Hospital of Guadalajara Fray Antonio Alcalde and can be requested with prior authorization. All data generated or analyzed during this study are included in this article. Further inquiries can be directed to the corresponding author.

## References

[1] Sriperumbuduri S, Welling P, Ruzicka M et al. Potassium and hypertension: a state-of-the-art review. Am J Hypertens 2024;37:91–100. 10.1093/ajh/hpad094 37772757

[2] Kovesdy CP, Appel LJ, Grams ME et al. Potassium homeostasis in health and disease: a scientific workshop cosponsored by the National Kidney Foundation and the American Society of Hypertension. J Am Soc Hypertens 2017;11:783–800. 10.1016/j.jash.2017.09.011 29030153

[3] Palmer BF, Clegg DJ. Hyperkalemia treatment standard. Nephrol Dial Transplant 2024;39:1097–104. 10.1093/ndt/gfae056 38425037

[4] Palmer BF, Clegg DJ. Hyperkalemia across the continuum of kidney function. Clin J Am Soc Nephrol 2018;13:155–7. 10.2215/CJN.09340817 29114006 PMC5753321

[5] Chávez-Íñiguez JS, Rifkin BS. Dual RAAS blockade in CKD: does the hype have teeth? Kidney360 2022;3:1277–80. 10.34067/KID.0000912022 35919530 PMC9337902

[6] Kanda E, Kashihara N, Kohsaka S et al. Clinical and economic burden of hyperkalemia: a nationwide hospital-based cohort study in Japan. Kidney Med 2020;2:742–52.e1. 10.1016/j.xkme.2020.09.003 33319198 PMC7729225

[7] Agiro A, Cook E, Mu F et al. Hyperkalemia and risk of CKD progression: a propensity score-matched analysis. Kidney360 2024;5:1824–34. 10.34067/KID.0000000000000541 39120948 PMC11687975

[8] de Rooij ENM, de Fijter JW, Le Cessie S et al. Serum potassium and risk of death or kidney replacement therapy in older people with CKD stages 4-5: eight-year follow-up. Am J Kidney Dis 2023;82:257–66.e1. 10.1053/j.ajkd.2023.03.008 37182596

[9] Kidney Disease: Improving Global Outcomes (KDIGO) CKD Work Group . KDIGO 2024 Clinical Practice Guideline for the Evaluation and Management of Chronic Kidney Disease. Kidney Int 2024;105:S117–314. 10.1016/j.kint.2023.10.018 38490803

[10] Chávez-Íñiguez JS, Maggiani-Aguilera P, Aranda-García de Quevedo A et al. Serum potassium trajectory during acute kidney injury and mortality risk. Nephron 2023;147:521–30. 10.1159/000529588 36808092

[11] Clase CM, Carrero JJ, Ellison DH et al. Potassium homeostasis and management of dyskalemia in kidney diseases: conclusions from a Kidney Disease: Improving Global Outcomes (KDIGO) Controversies Conference. Kidney Int 2020;97:42–61. 10.1016/j.kint.2019.09.018 31706619

[12] Flythe JE, Watnick S. Dialysis for chronic kidney failure: a review. JAMA 2024;332:1559–73. 10.1001/jama.2024.16338 39356511

[13] Elliott AB, Soliman KMM, Ullian ME. Hyperkalemia in chronic peritoneal dialysis patients. Ren Fail 2022;44:217–23. 10.1080/0886022X.2022.2032151 35166182 PMC8856104

[14] Álvarez-Rodríguez E, Olaizola Mendibil A, San Martín Díez MLÁ et al. Recommendations for the management of hyperkalemia in the emergency department. Emergencias 2022;34:287–97. 35833768

[15] García-García G, Ibarra-Estrada M, Perl J et al. Outcomes of initiating peritoneal dialysis versus hemodialysis in severe, symptomatic kidney failure. Kidney360 2025;6:1373–83. 10.34067/KID.0000000863 40694419 PMC12407124

[16] Sarafidis PA, Blacklock R, Wood E et al. Prevalence and factors associated with hyperkalemia in predialysis patients followed in a low-clearance clinic. Clin J Am Soc Nephrol 2012;7:1234–41. 10.2215/CJN.01150112 22595825 PMC3408123

[17] Gasparini A, Evans M, Barany P et al. Plasma potassium ranges associated with mortality across stages of chronic kidney disease: the Stockholm CREAtinine Measurements (SCREAM) project. Nephrol Dial Transplant 2019;34:1534–41. 10.1093/ndt/gfy249 30085251 PMC6735645

[18] Auguste BL, Bargman JM. Peritoneal dialysis prescription and adequacy in clinical practice: core curriculum 2023. Am J Kidney Dis 2023;81:100–9. 10.1053/j.ajkd.2022.07.004 36208963

[19] Maggiani-Aguilera P, Chávez-Iñiguez JS, Navarro-Gallardo JG et al. The impact of anatomical variables on haemodialysis tunnelled catheter replacement without fluoroscopy. Nephrology 2021;26:824–32. 10.1111/nep.13909 34081379

[20] Liu Y, Zou W, Wu J et al. Comparison between incremental and thrice-weekly haemodialysis: systematic review and meta-analysis. Nephrology 2019;24:438–44. 10.1111/nep.13252 29532551

[21] Chávez-Iñiguez JS, Maggiani-Aguilera P, Cruz-Ramos JA et al. Rectus abdominis muscle thickness as a predictor of peritoneal catheter dysfunction in emergency-start peritoneal dialysis patients. Clin Nephrol 2021;96:29–35. 10.5414/CN110075 33749580

[22] Nardelli L, Scalamogna A, Cicero E et al. Relationship between number of daily exchanges at CAPD start with clinical outcomes. Perit Dial Int 2024;44:98–108. 10.1177/08968608231209849 38115700

[23] Flanigan M, Gokal R. Peritoneal catheters and exit-site practices toward optimum peritoneal access: a review of current developments. Perit Dial Int 2005;25:132–9. 10.1177/089686080502500204 15796138

[24] von Elm E, Altman DG, Egger M et al.; STROBE Initiative. The Strengthening the Reporting of Observational Studies in Epidemiology (STROBE) statement: guidelines for reporting observational studies. Int J Surg 2014;12:1495–9. 10.1016/j.ijsu.2014.07.013 25046131

[25] Benchimol EI, Smeeth L, Guttmann A et al. The REporting of studies Conducted using Observational Routinely-collected health Data (RECORD) statement. PLoS Med 2015;12:e1001885. 10.1371/journal.pmed.1001885 26440803 PMC4595218

[26] Virtanen J, Heiro M, Koivuviita N et al. Survival, cumulative hospital days and infectious complications in urgent-start PD compared with urgent-start HD. Perit Dial Int 2025;45:224. 10.1177/08968608241244939 38661183

[27] Cho S, Lee YJ, Kim SR. Acute peritoneal dialysis in patients with acute kidney injury. Perit Dial Int 2017;37:529–34. 10.3747/pdi.2016.00264 28348102

[28] Gabriel DP, Caramori JT, Martim LC et al. High volume peritoneal dialysis vs daily hemodialysis: a randomized, controlled trial in patients with acute kidney injury. Kidney Int 2008;73:S87–93. 10.1038/sj.ki.5002608 18379555

[29] Ponce D, Berbel MN, Abrão JM et al. A randomized clinical trial of high volume peritoneal dialysis versus extended daily hemodialysis for acute kidney injury patients. Int Urol Nephrol 2013;45:869–78. 10.1007/s11255-012-0301-2 23065432

[30] Al-Hwiesh A, Abdul-Rahman I, Finkelstein F et al. Acute kidney injury in critically ill patients: a prospective randomized study of tidal peritoneal dialysis versus continuous renal replacement therapy. Ther Apher Dial 2018;22:371–9. 10.1111/1744-9987.12660 29575788

[31] Chávez-Iñiguez JS, Camacho-Guerrero JR, Ponce D. Peritoneal dialysis in acute kidney injury: your questions answered. Perit Dial Int 2025;45:265. 10.1177/08968608251344356 40400384

[32] Parapiboon W, Jamratpan T. Intensive versus minimal standard dosage for peritoneal dialysis in acute kidney injury: a randomized pilot study. Perit Dial Int 2017;37:523–8. 10.3747/pdi.2016.00260 28546367

[33] Pirklbauer M . Hemodialysis treatment in patients with severe electrolyte disorders: management of hyperkalemia and hyponatremia. Hemodial Int 2020;24:282–9. 10.1111/hdi.12845 32436307 PMC7496587

[34] Xu Q, Xu F, Fan L et al. Serum potassium levels and its variability in incident peritoneal dialysis patients: associations with mortality. PLoS One 2014;9:e86750. 10.1371/journal.pone.0086750 24475176 PMC3903570

[35] Eriguchi R, Obi Y, Soohoo M et al. Racial and ethnic differences in mortality associated with serum potassium in incident peritoneal dialysis patients. Am J Nephrol 2019;50:361–9. 10.1159/000502998 31522173 PMC6856395

[36] Xue C, Zhou C, Yang B et al. A nomogram to identify hyperkalemia risk in patients with advanced CKD. Kidney360 2022;3:1699–709. 10.34067/KID.0004752022 36514723 PMC9717672

[37] Montford JR, Linas S. How dangerous is hyperkalemia? J Am Soc Nephrol 2017;28:3155–65. 10.1681/ASN.2016121344 28778861 PMC5661285

